# Epoxide Hydrolase Family: Biological Functions, Disease Mechanisms, and Emerging Therapeutic Strategies

**DOI:** 10.3390/biology15090691

**Published:** 2026-04-28

**Authors:** Yadan Tan, Jingjing Xu, Ziteng Huang, Xiran Wang, Jinshan Xing, Shengbiao Li, Jingyan Yi

**Affiliations:** 1 Department of Medical Cell Biology and Genetics, School of Basic Medical Sciences, Southwest Medical University, Luzhou 646000, China; 2 Department of Anesthesiology, The Affiliated Hospital, Southwest Medical University, Luzhou 646000, China; 3 Key Laboratory of Medical Electrophysiology, Ministry of Education & Medical Electrophysiological Key Laboratory of Sichuan Province, Collaborative Innovation Center for Prevention of Cardiovascular Diseases, Institute of Cardiovascular Research, Southwest Medical University, Luzhou 646000, China; 4 Department of Neurosurgery, The Affiliated Traditional Chinese Medicine Hospital, Southwest Medical University, Luzhou 646000, China; 5 Department of Medical Cell Biology and Genetics, School of Basic Medical Sciences, Key Laboratory of Medical Electrophysiology, Ministry of Education, Institute of Cardiovascular Research, Southwest Medical University, Luzhou 646000, China; 6 Department of Medical Cell Biology and Genetics, School of Basic Medical Sciences, Basic Medicine Research Innovation Center for Cardiometabolic Diseases, Ministry of Education, Key Laboratory of Medical Electrophysiology, Ministry of Education, Institute of Cardiovascular Research, Southwest Medical University, Luzhou 646000, China

**Keywords:** epoxide hydrolases (EHs), lipid metabolism, inflammation, cardiovascular disease, therapeutic targets

## Abstract

Epoxide hydrolases (EHs) constitute a conserved enzyme family that catalyzes the hydrolysis of epoxides into less reactive diols. Beyond their canonical roles in xenobiotic detoxification, EHs have emerged as critical regulators of lipid metabolism, redox balance, and inflammatory signaling. Accumulating evidence implicates EH family members, particularly Ephx1 (microsomal EH) and Ephx2 (soluble EH), in cardiovascular diseases, cancer, neurodegeneration, metabolic disorders, and other pathological conditions. More recently, studies have uncovered specialized functions of Ephx3 and Ephx4, broadening our understanding of EH biology and highlighting their tissue-specific roles in skin homeostasis and lipid signaling. Here, we systematically review the structural features, catalytic mechanisms, and physiological functions of EHs, with an emphasis on their regulatory networks in human diseases. We further discuss advances in genetic, epigenetic, and translational studies that connect EHs to disease susceptibility and progression. Finally, we evaluate the therapeutic potential and challenges of targeting EHs, particularly soluble EH inhibitors, and propose future research directions to bridge basic discoveries with clinical translation. This review aims to provide a comprehensive framework for understanding the multifaceted roles of EHs and to inspire novel strategies for precision medicine.

## Simple Summary

Epoxide hydrolases (EHs) are a family of enzymes that catalyze the hydrolysis of epoxides to their corresponding and more stable diols. Initially, they were primarily recognized for their role in the detoxification metabolism of xenobiotic compounds. Recent studies have revealed that EH family members are also extensively involved in the metabolism and signaling regulation of endogenous lipids, thereby influencing a variety of biological processes including inflammatory responses, oxidative stress, autophagy, and cell survival. The mammalian EH family mainly comprises four members: Ephx1, Ephx2, Ephx3, and Ephx4, which exhibit distinct differences in tissue distribution and biological functions. Accumulating evidence indicates that EH dysfunction is closely associated with the pathogenesis and progression of numerous diseases, such as cardiovascular diseases, metabolic disorders, cancer, and neurological disorders. Among them, soluble epoxide hydrolase (sEH) has emerged as a significant potential therapeutic target, with several of its inhibitors currently under clinical or preclinical investigation. This review systematically summarizes the structural characteristics, regulatory mechanisms, and biological functions of EH family members. Furthermore, it aims to provide a comprehensive perspective on their roles in human diseases and the research progress regarding their potential as therapeutic targets.

## 1. Introduction

Epoxide hydrolases (EHs) are ubiquitous enzymes present in plants, animals, and microorganisms, encoded by genes that are widely conserved across genomes [1]. Initially identified as detoxifying enzymes [2], EHs catalyze the hydrolysis of reactive epoxides into more stable dihydrodiols, a function that underpins their potential in industrial and biocatalytic applications [3]. Beyond detoxification, however, EHs have emerged as key regulators of lipid and xenobiotic metabolism, implicating them in diverse physiological and pathological contexts.

The mammalian EH family is represented by four members—Ephx1, Ephx2, Ephx3, and Ephx4—each with distinct tissue distribution and biological roles. Human Ephx1 cDNA was first isolated in 1988 [4], marking the beginning of mechanistic studies on its detoxifying functions. In 1993 [5], Ephx2 cDNA was successfully cloned, which facilitated investigations into its roles in lipid metabolism and cardiovascular biology. In 2012, Decker and colleagues identified a highly conserved 16-amino acid motif, initially annotated as ABHD7 and ABHD9 in genomic databases, later renamed as EH3 (Ephx3) and EH4 (Ephx4) [6], respectively.

Despite accumulating evidence that EHs influence disease pathways ranging from cancer to cardiovascular disorders, knowledge of their molecular mechanisms remains fragmented. Here, we provide a framework that integrates the current understanding of EH structure, regulation, and function, with emphasis on post-translational modifications, cellular fate, and downstream targets. We also highlight their emerging roles in human disease, aiming to inform both mechanistic exploration and therapeutic innovation.

## 2. Structure and Localization of EH-Encoding Genes

Most members of the EH family belong to the α/β-hydrolase fold (ABHD) superfamily, which mediates lipid metabolism and signaling [7], although some lack this canonical feature [8]. Mechanistically, EHs catalyze the hydrolysis of epoxides through an aspartate-driven nucleophilic attack and substrate recognition by a tyrosine-containing cap domain. Together, EH3 and EH4 form a newly defined EH subfamily alongside mEH (Ephx1) and sEH (Ephx2), with emerging roles in lipid metabolism and cellular signaling. Indeed, EHs modulate the epoxy fatty acid (EpFA) levels [9], thereby influencing signaling pathways essential for homeostasis. Notably, knockout models reveal that the deletion of individual Ephx genes does not trigger the compensatory upregulation of other family members, underscoring their distinct biological roles [10].

### 2.1. Epoxide Hydrolase 1 (Ephx1)

Ephx1 spans ~35 kb [11] on chromosome 1 and encodes a 455-amino acid protein organized into nine exons. Its promoter and coding region harbor SNPs linked to interindividual differences in expression. Ephx1 is broadly expressed, with high abundance in liver, adipose tissue, adrenal gland, and placent [12,13]. The encoded protein, microsomal EH (mEH, ~52 kDa), localizes to the endoplasmic reticulum [12] and is enriched in hepatocytes surrounding central veins [14]. Functionally, mEH detoxifies xenobiotic epoxides [14,15,16], a role that extends to pulmonary metabolism where it helps limit inhaled toxicants [17].

### 2.2. Epoxide Hydrolase 2 (Ephx2)

Ephx2 (~45 kb, chromosome 8 [18,19]) encodes soluble EH (sEH), a 555-amino acid enzyme of ~62 kDa comprising a C-terminal epoxide hydrolase domain and an N-terminal phosphatase domain connected by a proline-rich linker. sEH is highly expressed in cardiomyocytes, where it hydrolyzes epoxy eicosatrienoic acids (EETs) and critically regulates post-ischemic recovery [20]. EETs are epoxy fatty acids derived from arachidonic acid. sEH catalyzes the hydrolysis of EETs into their corresponding diols, namely dihydroxyeicosatrienoic acids (DHETs) [21,22,23], thereby reducing the epoxide-to-diol ratio (the ratio between cytochrome P450-derived epoxy fatty acids and their corresponding diols generated via sEH-mediated hydrolysis is commonly used to reflect epoxide hydrolase activity) and reshaping downstream signaling pathways. Its expression is dynamically regulated in cancer: downregulated in hepatocellular carcinoma but upregulated in seminoma, cholangiocarcinoma, and advanced ovarian cancer [24]. Elevated sEH has also been associated with hippocampal atrophy in Alzheimer’s disease (AD) [25,26,27], though causality remains unresolved. At the molecular level, sEH hydrolyzes epoxylipids to less active diols, influencing inflammation, oxidative stress, and atherosclerosis. Genetic variants alter enzyme activity and may shape disease susceptibility [28]. Distribution studies place sEH in hepatocytes [29,30], renal microvasculature, proximal tubules [31,32], and specific neuronal and glial populations, with activity increasing with age and inversely correlating with circulating EET levels [33].

### 2.3. Epoxide Hydrolase 3 (Ephx3)

Ephx3 (also known as ABHD9, ~27 kb, chromosome 19) encodes a 360-amino acid protein (~41 kDa) localized to the plasma membrane. Expression is enriched in skin, lymphoid tissue, bone marrow, and the proximal gut [6], with lower levels in skeletal muscle and heart [34]. EH3 has been shown to metabolize EETs and modulate angiogenesis, although its enzymatic activity is thermolabile [33]. Notably, Ephx3 knockout models do not exhibit an overt systemic phenotype; however, Ephx3 deficiency has been reported to reduce the hydrolysis of ceramide linoleate epoxides and impair skin barrier function [33], suggesting functional redundancy with other EHs.

### 2.4. Epoxide Hydrolase 4 (Ephx4)

Ephx4 (ABHD7, ~39 kb, chromosome 1) encodes a 362-amino acid protein (~42 kDa) with nuclear localization. It is predominantly expressed in the brain, although its expression is not restricted to this tissue; it has also been detected in other tissues, such as colonic epithelium, as well as in tumor samples [35]. EH4 remains the only member of the EH family for which epoxide hydrolase activity has not been definitively established. Despite sharing sequence and structural homology with other EH family members, its enzymatic function remains unclear. Importantly, Ephx4 (also known as ABHD7) has been identified as a direct transcriptional target of the oncogene ZNF217, implicating its dysregulation in colorectal and other cancers [6].

## 3. EH-Mediated Post-Translational Modifications

**Figure 1:**
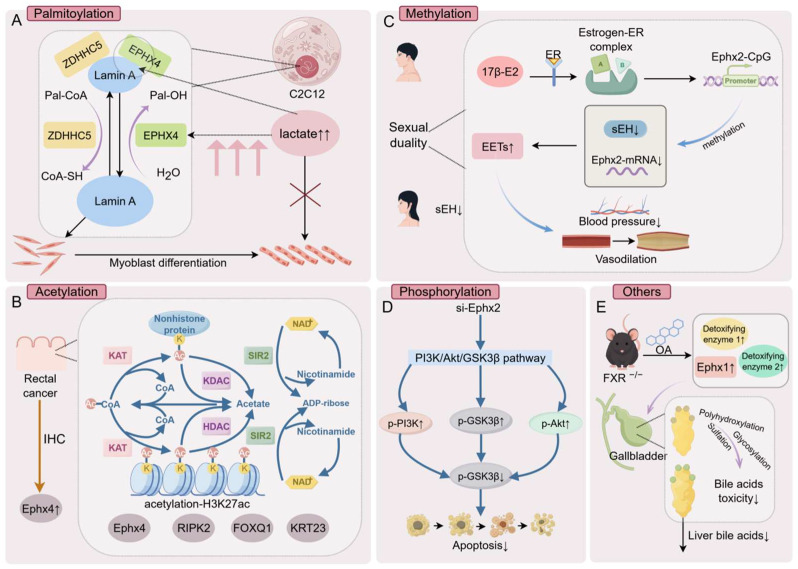
Post-translational modifications mediated by EHs. (**A**). Under normal lactate conditions, myocyte differentiation proceeds normally. Elevated lactate levels promotes the palmitoylation of lamin A by inhibiting the depalmitoylase ABHD7 (EPHX4), thereby regulating myocyte differentiation. (**B**). Ephx4 is activated and highly expressed in colorectal cancer due to H3K27ac modification, suggesting its significant role in tumorigenesis. (**C**). Estrogen induces the methylation of the Ephx2 promoter via ER, suppressing its expression, which may explain the protective effects observed in females during ischemic events in the heart and brain. (**D**). Ephx2 regulates the phosphorylation status of the PI3K/Akt/GSK3β signaling pathway, playing a pivotal role in oxidative stress response and apoptosis regulation. (**E**). Ephx1 plays a crucial role in bile acid metabolism and detoxification processes in diseases associated with cholestasis.

Post-translational modifications (PTMs) fine-tune protein structure and function, enabling dynamic regulation of signaling, metabolism, and gene expression. Common PTMs—including palmitoylation, acetylation, methylation, and phosphorylation—are increasingly recognized as critical layers of EH regulation, linking enzymatic activity to diverse physiological and pathological processes (Figure 1).

Palmitoylation is a reversible lipid modification that attaches palmitic acid to cysteine residues, altering protein membrane association and localization. ABHD7 (also known as EPHX4/EPHXRP [36]), a brain-restricted epoxide hydrolase family member, has been identified as a depalmitoylase involved in muscle cell differentiation, highlighting a direct role of EHs in PTMs. In HEK293T and C2C12 cells, ABHD7 interacts with lamin A within the nucleus and selectively removes its palmitoylation. The palmitoylation of lamin A is dynamically regulated by the DHHC-type palmitoyltransferase 5 (ZDHHC5) and the depalmitoylating enzyme α/β hydrolase domain-containing protein 7 (ABHD7) [36]. Loss of ABHD7 increases lamin A palmitoylation, establishing ABHD7 as its primary depalmitoylase. Intriguingly, lactate disrupts ABHD7–lamin A binding, thereby enhancing lamin A palmitoylation, suggesting a direct link between cellular metabolism and nuclear protein modification [36].

In addition to direct enzymatic regulation of PTMs, EHs may also influence gene expression indirectly through broader modification networks. Accordingly, the following sections focus on epigenetic and post-translational mechanisms that regulate EH expression and function.

### 3.1. Acetylation

Lysine acetylation regulates protein conformation, activity, and chromatin accessibility. In colorectal cancer, elevated H3K27 acetylation (H3K27ac) correlates with oncogene activation. Genome-wide ChIP-seq revealed enrichment of H3K27ac at Ephx4 loci in tumor tissue, consistent with immunohistochemistry demonstrating elevated Ephx4 protein expression. These findings place Ephx4 within the epigenetic landscape of colorectal tumorigenesis, highlighting acetylation-driven transcriptional activation as a regulatory axis [37].

### 3.2. Methylation

Methylation exerts profound regulatory effects on EH gene expression and tumor biology. Hypermethylation of the Ephx2 promoter leads to transcriptional silencing of soluble epoxide hydrolase (sEH), mediated by estrogen receptor (ER)-dependent recruitment of DNA methyltransferases to CpG-rich regions [38]. The estrogen–ER complex refers to the ligand–receptor complex formed upon the binding of estrogen to the estrogen receptor (ER). Its principal ligand is the natural estrogen 17β-estradiol (17β-E2), the most potent and predominant estrogen in vivo. Upon the binding of 17β-estradiol (17β-E2), ER forms a ligand–receptor complex that translocates into the nucleus and binds to estrogen response elements (EREs) within target gene promoters. In this context, ER recruits DNA methyltransferases (e.g., DNMT1 and DNMT3A/3B) to the Ephx2 promoter, promoting local DNA methylation and suppressing transcription. Beyond its canonical role as a transcription factor, ER also functions as an epigenetic scaffold, coordinating interactions with chromatin remodeling complexes to stabilize gene silencing [38,39]. Given the marked sex differences in circulating estrogen levels, this ER-driven epigenetic mechanism contributes to sex-biased sEH [40,41] expression and may underlie the relative protection observed in females against ischemic cardiovascular and neurological injury [38,40,41]. Aberrant DNA methylation is a hallmark of tumor epigenetics. In head and neck squamous cell carcinoma (HNSCC), differential methylation of Ephx3 suggests that EH family members participate in tumor-associated epigenetic regulatory networks. Clinically, Ephx3 methylation also exhibits prognostic value: hypomethylation and high expression correlate with improved survival in HNSCC, whereas hypermethylation is associated with poor outcomes in oral squamous cell carcinoma, supporting its potential as a prognostic biomarker [42,43].

### 3.3. Phosphorylation

Phosphorylation represents a key mechanism linking EH activity to intracellular signaling pathways. EHs regulate lipid signaling homeostasis, with sEH playing a central role in the metabolism of epoxy fatty acids such as EETs. Previous studies have shown that silencing Ephx2 reduces sEH expression, thereby decreasing the degradation of cytoprotective epoxy fatty acids and increasing their intracellular levels. The silencing of Ephx2 reduces sEH activity, leading to decreased hydrolysis and consequent accumulation of EETs. These bioactive lipid mediators function as signaling molecules that activate membrane-associated receptors or upstream kinases, thereby promoting the activation of the PI3K/Akt pathway [44]. Concurrently, this intervention markedly enhances the phosphorylation of key signaling proteins, including PI3K, Akt, and GSK3β. Mechanistically, increased EET levels enhance the phosphorylation of PI3K, which in turn activates Akt through site-specific phosphorylation. Activated Akt subsequently phosphorylates downstream targets such as GSK3β (typically at Ser9), resulting in the inhibition of its pro-apoptotic activity [44]. Notably, the phosphorylation of GSK3β suppresses its pro-apoptotic activity and promotes cell survival, thereby facilitating the activation of the PI3K/Akt/GSK3β signaling pathway and further increasing the phosphorylation levels of downstream effectors [44]. Consistently, Ephx2 silencing markedly increases the phosphorylation levels of PI3K, Akt, and GSK3β, ultimately alleviating oxidative stress-induced cellular injury. Collectively, these findings position sEH as a critical nodal regulator linking EH-mediated lipid metabolism to oxidative stress responses and apoptosis control [44]. Ultimately, these effects alleviate oxidative stress-induced cellular injury and inhibit apoptosis. Collectively, sEH is considered a critical nodal molecule linking EH activity with oxidative stress responses and the regulation of apoptosis.

### 3.4. Other Modifications

EHs also participate in bile acid detoxification pathways. The farnesoid X receptor (FXR) is a bile acid-sensing nuclear receptor that regulates bile acid synthesis, transport, and metabolic homeostasis [45]. The disruption of FXR signaling leads to the reprogramming of hepatic detoxification networks, particularly those involved in oxidative and conjugative metabolism [45]. In FXR-deficient mice, Ephx1 expression is markedly upregulated, particularly under oleanolic acid treatment [46]. As part of the detoxification machinery, microsomal epoxide hydrolase (mEH) catalyzes the hydrolysis of reactive epoxide intermediates into corresponding diols, thereby altering the chemical structure and polarity of bile acid derivatives and facilitating their subsequent metabolism. These modified bile acids are further processed through phase I and phase II detoxification pathways, including cytochrome P450-mediated hydroxylation and conjugation by sulfotransferases (SULTs) and UDP-glucuronosyltransferases (UGTs). Such reactions increase hydrophilicity, promote biliary excretion, and reduce intracellular accumulation of cytotoxic hydrophobic bile acids, ultimately alleviating cholestatic liver injury [45,46]. This positions Ephx1 as a metabolic safeguard in bile acid homeostasis and a potential therapeutic target in cholestatic liver disease.

## 4. Biological Functions Regulated by EHs

### 4.1. Inflammation

Lipids are central to inflammation, functioning not only as metabolic fuels but also as bioactive mediators that shape immune responses. Epoxylipids, in particular, act as potent regulators of immune cell function and inflammatory tone. In adipocytes lacking autophagy (Atg7 deficiency), Ephx1 is upregulated via NRF2, disturbing oxylipid balance and suppressing IL-10 secretion, thereby exacerbating intestinal inflammation [47]. sEH likewise drives inflammation by converting anti-inflammatory EETs into pro-inflammatory DHETs. Environmental exposures, such as prometryn, elevate sEH activity and oxidized lipid levels in both mice and humans, aggravating vascular dysfunction [48]. High-fat diet models reveal that adipose–liver crosstalk amplifies inflammation through sEH induction in the liver, while pharmacologic sEH inhibition mitigates steatohepatitis and adipose inflammation [49]. Resolvin E1 (RvE1) is a specialized pro-resolving lipid mediator that actively promotes the resolution of inflammation and the restoration of tissue homeostasis. Accumulating evidence indicates that RvE1 significantly attenuates vascular inflammation and inhibits atherosclerotic plaque formation during the development of atherosclerosis (AS), and these protective effects occur independently of cholesterol lowering while also exhibiting synergistic effects with statins. Emerging evidence suggests that EPHX4 is functionally linked to EET metabolism and may participate in the regulation of inflammation and atherosclerosis through the modulation of EET levels. However, its precise substrate specificity and enzymatic activity remain incompletely characterized. Importantly, RvE1 has been shown to significantly reduce circulating EPHX4 levels. This reduction is proposed to enhance the pro-resolving effects of EETs [50,51], thereby contributing to the attenuation of vascular inflammation and the progression of atherosclerosis [52].

### 4.2. Autophagy

Autophagy maintains cellular homeostasis through lysosomal recycling of damaged proteins and organelles, while also regulating lipid balance and inflammatory responses. Autophagy plays a critical role in EH-related signaling and influences cellular metabolic homeostasis and disease progression through multilayered mechanisms. In laryngeal carcinoma, integrated bioinformatic and experimental analyses have identified Ephx3 as an autophagy-related prognostic gene, with its expression closely associated with mitophagy activity [53]. Specifically, mitochondrial damage triggers mitophagy, enabling the selective clearance of dysfunctional mitochondria and thereby modulating mitochondrial function and cellular energy metabolism; alterations in Ephx3 expression are linked to this process and may influence tumor metabolic adaptation and patient prognosis.

In visceral adipose tissue, the deficiency of adipocyte autophagy elevates oxidative stress and activates NRF2 signaling, thereby inducing the NRF2–Ephx1 regulatory axis [47]. As a key transcription factor in oxidative stress responses, NRF2 directly binds to the Ephx1 promoter and enhances its transcription, promoting epoxy lipid metabolism and altering the balance of oxidized lipids. Meanwhile, although Ephx2 mRNA levels remain largely unchanged, its protein expression is significantly increased, suggesting regulation via post-transcriptional mechanisms such as enhanced translation efficiency or protein stability. In models of renal injury, decreased sEH expression is closely associated with enhanced autophagic activity [52]. Mechanistically, the downregulation of sEH promotes the expression and activation of autophagy-related proteins, including Beclin-1, LC3, and Atg family members, thereby facilitating autophagosome formation and enhancing the degradation and recycling of damaged cellular components, ultimately contributing to tissue repair. Collectively, sEH is considered a negative regulator of autophagy, and its suppression may relieve inhibitory constraints on autophagic processes and enhance autophagy-dependent tissue repair capacity.

### 4.3. Oxidative Stress and Cellular Senescence

**Figure 2:**
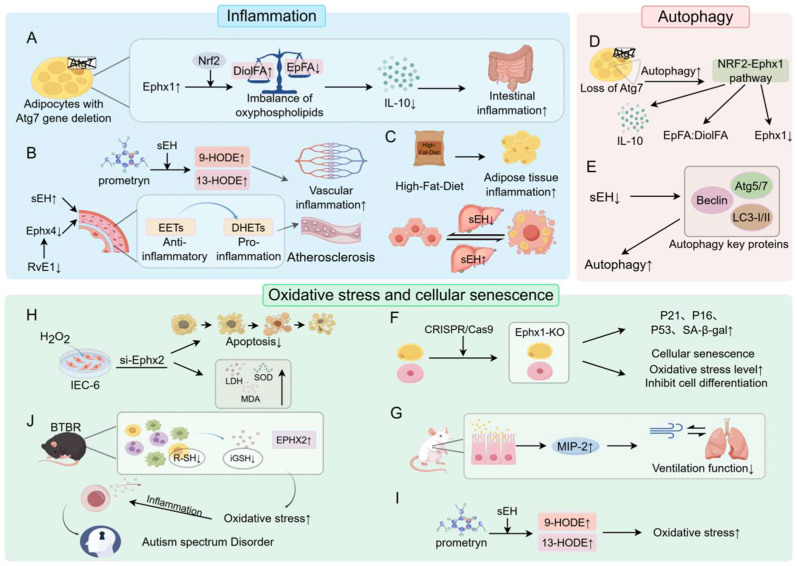
Roles of EHs in inflammation, autophagy, and oxidative stress. (**A**) Atg7 deletion disrupts adipocyte homeostasis and exacerbates inflammation. (**B**) EHs promote inflammation and affect vascular function by modulating oxidized lipid levels. (**C**) High-fat diets are closely linked to adipose tissue inflammation. The expression level of sEH is positively correlated with the degree of hepatocyte steatosis, and inhibition of sEH can alleviate lipid accumulation in hepatocytes. (**D**) The NRF2–Ephx1 axis is implicated in the regulation of autophagy. (**E**) sEH modulates autophagy to exert protective effects in kidney injury repair. (**F**) Elevated EPHX1 levels exacerbate oxidative stress and induce cellular senescence features. (**G**) Ephx1 plays a key role in the maintenance of respiratory function. (**H**) Ephx2 mitigates oxidative stress-induced damage. (**I**) Prometryn exposure intensifies oxidative stress. (**J**) The pathogenesis of autism spectrum disorder (ASD) may be associated with elevated EPHX2 levels, which induce oxidative stress.

Oxidative stress, driven by excess reactive oxygen species (ROS), damages macromolecules and contributes to senescence, a stable growth-arrested state linked to aging and disease. Loss of Ephx1 suppresses adipocyte differentiation, blunts insulin responsiveness, and accelerates senescence, marked by P21/P16 upregulation, SA-β-gal activity, and phosphorylated p53 accumulation [48]. In adipose stem cells, Ephx1 deletion similarly promotes senescence and differentiation defects. Environmental [48] insults amplify this process: prometryn exposure increases mEH and sEH activity, elevating oxidized lipids such as 9-HODE and 13-HODE, consistent with heightened oxidative stress. Functionally, Ephx1 polymorphisms associate with impaired lung function following particulate exposure [54]. In rat lung epithelial cell models, fine particulate matter exposure elevates MIP-2 levels, leading to a more pronounced decline in respiratory function in individuals with specific Ephx1-related genotypes, thereby implicating EH variants in susceptibility to environmentally induced oxidative injury. Conversely, Ephx2 silencing attenuates H_2_O_2_-induced ROS accumulation, preserves mitochondrial membrane potential, and protects against apoptosis [44], highlighting its role in stress resilience. Finally, in neurodevelopmental disorders, oxidative stress intersects with immune dysregulation: in the BTBR mouse model of autism spectrum disorder, immune cells show reduced thiol and glutathione levels alongside increased EPHX2 expression [55], linking EH dysregulation to redox imbalance and pro-inflammatory immune states (Figure 2).

### 4.4. Cell Proliferation and Apoptosis

Epoxide hydrolases shape tumor biology by regulating EpFA turnover and downstream signaling. Ephx1 polymorphisms alter cancer susceptibility by modifying EpFA hydrolysis, impacting angiogenesis and proliferation, and ultimately influencing tumor progression [56]. mEH is often elevated in hepatocellular carcinoma and breast cancer [57,58], suggesting a role in restraining growth and metastasis. Conversely, sEH can either promote tumor expansion through EET hydrolysis or show reduced expression in tumor cells [59,60], reflecting context-specific effects. In castration-resistant prostate cancer, Ephx1 is upregulated, while miR-491-5p directly targets its 3’UTR to suppress proliferation and migration, highlighting a post-transcriptional checkpoint [61]. For sEH, the balance of EETs is decisive: the inhibition of Ephx2 stabilizes EETs and accelerates tumor growth and metastasis, whereas sEH overexpression or EET antagonism limits progression [59]. In colorectal cancer, enforced Ephx2 expression suppresses invasion and triggers apoptosis, an effect reversed by siRNA knockdown [62]. Together, these findings reveal EHs as double-edged regulators of tumor growth, with context dictating whether they act as oncogenic drivers or tumor suppressors.

## 5. EH Family in Human Disease

### 5.1. Cancers

EH family members are frequently dysregulated in malignancies, where they influence tumor metabolism, redox balance, and immune modulation. Accumulating evidence reveals isoform- and tissue-specific roles that may serve as biomarkers or therapeutic targets (Table 1).

#### 5.1.1. Laryngeal Cancer

Ephx4 is markedly upregulated in laryngeal squamous cell carcinoma (LSCC), and its high expression is associated with unfavorable prognosis [63]. Analysis of TCGA datasets shows elevated Ephx4 mRNA in LSCC, and patients with lower expression display longer survival. Computational deconvolution of immune infiltration landscapes further indicates that Ephx4 levels correlate with changes in immune cell composition. Functionally, the knockdown of Ephx4 suppresses LSCC proliferation and migration, whereas its overexpression restores these phenotypes. Immunohistochemistry confirms increased Ephx4 protein levels in tumor tissues, supporting a role in tumor progression and as a potential prognostic marker.

#### 5.1.2. Renal and Liver Tumors

Genes involved in oxidative stress responses, including Ephx4, are consistently overexpressed in renal clear cell carcinoma, chromophobe renal carcinoma, and hepatocellular carcinoma [64]. These expression changes suggest a broader role for Ephx4 in redox-related tumor biology and highlight its potential as both a prognostic biomarker and a therapeutic target.

#### 5.1.3. Colorectal Cancer

In colorectal tumors, Ephx2 is significantly downregulated [62]. As Ephx2 participates in peroxisomal fatty acid degradation and promotes apoptosis through ROS accumulation, its loss facilitates tumor progression. Clinical correlations further link reduced Ephx2 with enhanced invasion and diminished apoptosis in colorectal cancer cells. In contrast, Ephx4 expression is elevated in colorectal carcinoma and in peritoneal mucinous tumors, with marked differences compared to normal colonic epithelium [35], implicating it in malignant transformation. A distinct pattern emerges where Ephx1 and Ephx2 are reduced, while Ephx3 and Ephx4 are elevated [65], pointing toward isoform-specific reprogramming during colorectal tumorigenesis.

#### 5.1.4. Head and Neck Squamous Cell Carcinoma

Aberrant regulation of Ephx3 is closely associated with patient outcomes in head and neck cancers. Low methylation combined with high expression predicts improved survival [66], while the miRNA-mediated downregulation of Ephx3 suppresses tumor cell proliferation [67]. These findings highlight both epigenetic and post-transcriptional regulation of EHs in driving tumor progression.

#### 5.1.5. Lung Cancer

Polymorphisms in Ephx1 (mEH) strongly influence lung cancer susceptibility [68]. The high-activity H139R variant increases metabolic activation of polycyclic aromatic hydrocarbons, leading to elevated DNA adduct formation and higher cancer risk [69,70], particularly in smokers. In contrast, the low-activity Y113H variant exerts variable effects, acting as either protective or neutral depending on exposure [71]. These observations underscore the dual roles of mEH activity in carcinogen detoxification versus activation [72,73,74,75].

#### 5.1.6. Prostate Cancer

Ephx2 is frequently deleted or downregulated in metastatic and recurrent prostate cancer [24]. Low Ephx2 mRNA levels correlate with shorter disease-free survival and earlier biochemical recurrence, indicating its value as a prognostic biomarker. Interestingly, the stable silencing of Ephx2 in prostate cancer cells does not significantly affect proliferation or response to antiandrogen therapy, suggesting that its clinical impact may be more prognostic than mechanistic in tumor growth.

#### 5.1.7. Other Cancers

Genetic and epigenetic alterations of EHs extend across diverse tumor types. Ephx1 polymorphisms are associated with risks of lung, esophageal, colorectal, prostate, bladder, and breast cancers [56,76,77]. Epigenetic silencing of ABHD9 is observed in melanoma, gastric cancer, salivary adenoid cystic carcinoma, and colorectal cancer, where it correlates with the recurrence or deregulation of B-cell malignancies. Mutations in ABHD9 also cause autosomal recessive congenital ichthyosis, illustrating its broader physiological relevance. In oral and pharyngeal tissues, elevated Ephx1 expression is linked to increased susceptibility to laryngeal, oral, and pharyngeal cancers [78].

### 5.2. Metabolic Diseases

EH family enzymes are increasingly recognized as key regulators of metabolic homeostasis, linking lipid signaling, oxidative stress, and inflammatory responses to the pathogenesis of diabetes, liver injury, and kidney disease.

#### 5.2.1. Diabetes

Loss-of-function mutations in Ephx1 cause lipoatrophic diabetes, characterized by severe adipose tissue deficiency, insulin resistance, and multisystem dysfunction [79], underscoring its essential role in metabolic regulation. In contrast, the genetic deletion or pharmacological inhibition of soluble epoxide hydrolase (sEH, encoded by Ephx2) lowers plasma glucose, enhances insulin receptor signaling, and improves systemic insulin sensitivity [80]. Ephx2^−/−^ mice exhibit enlarged islets, enhanced vascularization, and elevated levels of EETs, which stabilize glycemic control. Under a high-fat diet challenge, these mice maintain improved insulin responsiveness and glucose tolerance. These findings suggest that the loss of sEH activity augments EET-mediated signaling to protect against insulin resistance, providing a rationale for targeting sEH in metabolic disease.

#### 5.2.2. Alcohol-Related Liver Disease (ALD)

sEH expression is closely associated with ethanol-induced liver injury. By regulating the conversion of epoxylipids to their corresponding diols and thereby altering the epoxide-to-diol ratio [81,82], sEH plays a critical role in the pathogenesis of ALD. Elevated sEH activity converts EETs into DHETs, reducing the epoxide-to-diol ratio and attenuating the anti-inflammatory, antioxidant, and hepatoprotective effects mediated by EETs. This shift promotes inflammation, lipid metabolic dysregulation, and hepatocellular injury. Conversely, the hepatocyte-specific deletion of sEH elevates EET levels while reducing DHET formation, significantly increasing the epoxide-to-diol ratio, thereby enhancing anti-inflammatory and cytoprotective signaling. The hepatocyte-specific deletion of Ephx2 significantly attenuates steatosis, inflammation, and hepatocellular damage in chronic-plus-binge ethanol feeding models [83], as indicated by reduced serum ALT levels. The absence of sEH dampens the hepatic expression of pro-inflammatory cytokines including TNF-α, IL-1β, and MCP-1, particularly within immune cell compartments. Mechanistically, the loss of sEH blunts the ethanol-induced phosphorylation of NF-κB signaling. Pharmacological inhibition with agents such as TPPU produces similar protective effects, mitigating ER stress, oxidative stress, and apoptosis, thereby preserving hepatic function. Collectively, these observations establish sEH as a central mediator of ALD pathogenesis and a promising therapeutic target.

#### 5.2.3. Renal Disease

**Figure 3:**
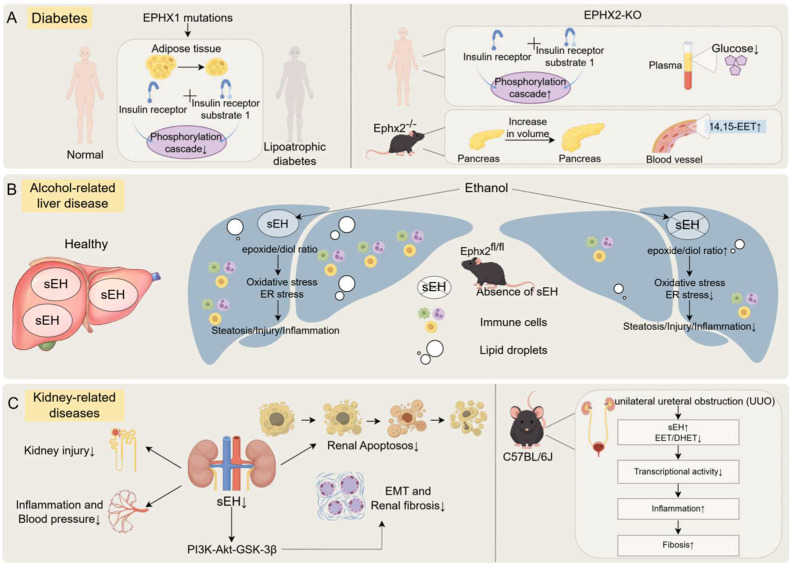
Roles of the EH family in metabolic diseases. (**A**) The EH family regulates glucose homeostasis and insulin signaling pathways through the modulation of EET levels, thus improving insulin resistance. (**B**) sEH deletion alleviates lipid droplet accumulation, oxidative stress, and inflammation in alcoholic liver disease. (**C**) sEH confers protective effects in various kidney injury models by reducing renal damage, lowering inflammation, improving renal function, and mitigating oxidative stress.

In kidney injury models, both genetic ablation and the pharmacological inhibition of sEH confer robust renoprotection [84]. Mice lacking sEH display reduced tubular injury, attenuated inflammation, improved renal function, and lower oxidative stress. Consistent effects are observed in models of acute kidney injury (AKI) and chronic kidney disease (CKD), where sEH inhibition alleviates oxidative stress, ER stress, and apoptosis, while promoting autophagy and tubular cell survival. sEH is predominantly expressed in proximal tubular cells and is upregulated in glomerulonephritis and obstructive nephropathy [85], implicating it in human renal pathophysiology. In diabetic and obstructive nephropathy, the inhibition of sEH increases intrarenal EET levels and prevents tubulointerstitial fibrosis and inflammation [86]. Mechanistic studies further show that sEH promotes epithelial–mesenchymal transition (EMT) by hydrolyzing renoprotective EETs into inactive dihydroxy eicosatrienoic acids (DHETs) [87]. Proteinuria-driven EMT is accompanied by the activation of PI3K–Akt–GSK3β signaling, which is reversed by sEH inhibition, restoring epithelial marker expression. Thus, targeting sEH represents a viable therapeutic strategy to counter EMT and fibrosis in progressive kidney disease (Figure 3).

### 5.3. Cardiovascular Disease

Although less extensively studied than sEH, mEH has emerged as a regulator of vascular and cardiac function. By hydrolyzing EpFA into less bioactive diols, mEH reduces the vasodilatory potential of EETs. In the brain, EETs act as potent dilators of cerebral arteries; enhanced hydrolysis of EETs in an mEH gain-of-function mutant (E404D) compromises vasodilation, directly linking mEH activity to cerebrovascular regulation. In the heart, mEH is highly expressed and contributes to EpFA turnover. While isolated mEH deficiency has minimal impact on ischemia-induced EpFA metabolism, the combined deletion of mEH and sEH markedly improves post-ischemic cardiac recovery, highlighting the cooperative roles of the two enzymes in myocardial contraction and adaptation to stress.

mEH also functions as a xenobiotic-metabolizing enzyme, hydrolyzing environmental epoxides and polycyclic aromatic hydrocarbons (PAHs) into diols that may promote atherosclerosis [88]. Genetic variants of Ephx1 show complex associations with vascular disease, including a modest reduction in diabetes-associated ischemic stroke risk. In contrast, Ephx2 polymorphisms generally increase susceptibility to ischemic stroke. Stratified analyses indicate that Ephx2 exacerbates hypertension-related stroke, whereas under hyperglycemic conditions, Ephx2 transcription is downregulated, yet its polymorphisms still increase diabetes-associated stroke risk. Complementary insights come from mouse models: double deletion of Ephx1 and Ephx2 enhances post-infarction functional recovery more effectively than the loss of Ephx2 alone [10], underscoring the utility of dual knockout models in probing EET-mediated cardioprotection. The cardiomyocyte-specific deletion of Ephx2 further indicates that sEH influences post-ischemic recovery predominantly through cardiomyocyte function rather than the modulation of coronary blood flow [20].

sEH has also been implicated in vascular calcification and remodeling. In models of adenine–phosphate-induced vascular calcification, the genetic deletion of Ephx2 suppresses vascular smooth muscle cell (VSMC) transdifferentiation and calcification [89]. Mechanistic analyses implicate the mitochondrial deacetylase SIRT3: sEH promotes SIRT3 degradation, whereas sEH deficiency preserves SIRT3 expression, thereby restraining VSMC calcification. These findings position sEH inhibition as a potential therapeutic avenue for preventing vascular calcification. Additional studies demonstrate that sEH deficiency or pharmacological inhibition attenuates neointimal formation [90], suggesting clinical relevance in smoking-related cardiovascular risk. Consistently, nicotine exposure enhances vascular sEH expression and activity, lowering EET levels and promoting arterial stiffening and extracellular matrix remodeling [91]. The loss of sEH mitigates these effects, partly by preserving SIRT1 signaling, thereby limiting nicotine-induced vascular stiffening.

Beyond vascular remodeling, sEH plays a central role in cardiometabolic dysfunction. In diet-induced lipotoxic cardiomyopathy, Ephx2 knockout (sEH-KO) mice show markedly reduced myocardial lipid accumulation and preserved cardiac function, independent of systemic lipid or blood pressure changes [92]. These findings implicate sEH as a driver of lipotoxic injury via direct effects on cardiomyocyte lipid handling. Similarly, in models of type 1 diabetes mellitus (T1DM), increased cardiac sEH expression is associated with the progression of diabetic cardiomyopathy [93], further highlighting its pathological role.

### 5.4. Brain Disorders

#### 5.4.1. Neurodegenerative Disease

Accumulating evidence implicates soluble epoxide hydrolase (sEH, encoded by Ephx2) in the pathogenesis of Alzheimer’s disease (AD). Genetic association analyses indicate a causal relationship between Ephx2 and AD, with the colocalization of Ephx2 variants and AD risk loci [25]. Notably, Ephx2 is associated with hippocampal volume, a key structural correlate of AD pathology. In experimental models, hepatic sEH activity increases with age, suggesting a liver–brain axis in AD [94]. The conditional deletion of Ephx2 in the liver of 3 × TgAD mice reduces cerebral Aβ burden, tau pathology, and cognitive deficits, accompanied by decreased tau phosphorylation. Mechanistically, hepatic sEH influences amyloid precursor protein (APP) processing by altering C-terminal fragments and BACE1 expression, thereby modulating Aβ homeostasis in the brain. Specifically, elevated sEH activity upregulates BACE1 expression [94], promoting the β-site cleavage of APP and increasing the generation of β-type C-terminal fragments. These fragments are subsequently processed by γ-secretase to produce Aβ, leading to enhanced amyloid accumulation. Conversely, the inhibition or deletion of sEH reduces BACE1 expression and the formation of APP β-cleavage C-terminal fragments, thereby suppressing Aβ production. These findings indicate that sEH influences AD pathology by regulating BACE1-mediated APP β-cleavage and C-terminal fragment generation, ultimately modulating Aβ metabolism.

In addition, elevated hepatic sEH activity reduces levels of 14,15-EET, a lipid mediator that promotes ApoE release from astrocytes and facilitates the TREM2-dependent microglial phagocytosis of Aβ. These findings establish hepatic sEH as a systemic regulator of AD pathology via the ApoE–TREM2 axis.

#### 5.4.2. Traumatic Brain Injury

Liver–brain crosstalk also emerges in traumatic brain injury (TBI). In controlled cortical impact (CCI) and closed-head injury (CHI) models, hepatic sEH activity undergoes dynamic changes [95]. The genetic ablation or knockdown of Ephx2 in the liver attenuates CCI-induced neuropathology and promotes functional recovery, whereas the hepatic overexpression of Ephx2 exacerbates injury outcomes. Mechanistically, the loss of hepatic sEH enhances the generation of neuroprotective A2 astrocytes in the injured cortex and increases the secretion of astrocyte-derived protective factors. Elevated circulating levels of 14,15-EET appear to mediate these effects, indicating that hepatic sEH modulates the systemic lipid milieu to influence brain repair processes.

#### 5.4.3. Depression

**Figure 4:**
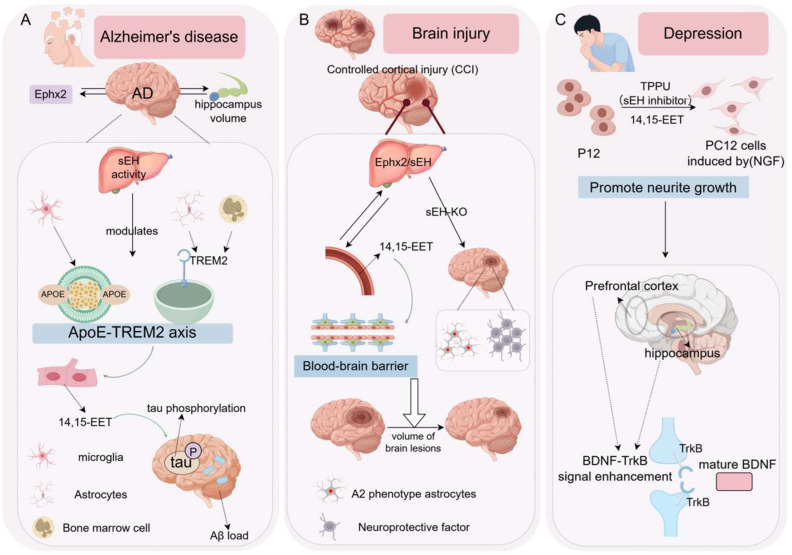
Roles of the EH family in neurological diseases. (**A**) Hepatic sEH activity regulates microglial Aβ clearance through the ApoE-TREM2 axis, playing a critical role in an Alzheimer’s disease mouse model. (**B**) Hepatic Ephx2 expression is closely associated with neurological recovery; its knockout enhances EET levels, improves blood-brain barrier integrity, promotes the polarization of astrocytes toward the A2 neuroprotective phenotype and the secretion of neuroprotective factors, and ultimately reduces brain lesion volume and ameliorates neurological function. (**C**) sEH plays a central role in inflammation and social stress models of depression; treatment with the sEH inhibitor TPPU rapidly prevents and reverses depressive-like behaviors while enhancing BDNF-TrkB signaling associated with neuronal plasticity.

sEH has also been implicated in mood regulation. Both patients with major depressive disorder and mouse models of stress-induced depression exhibit increased sEH expression in the brain [96]. Functional studies demonstrate that sEH activity contributes to depressive-like behavior in inflammatory and social defeat stress paradigms. Pharmacological inhibition with TPPU exerts rapid antidepressant effects, preventing and reversing behavioral phenotypes while enhancing BDNF–TrkB signaling and neuronal plasticity. Consistently, Ephx2 knockout mice display resilience to repeated social defeat, supporting a role for sEH in stress adaptation via the regulation of neurotrophic signaling pathways (Figure 4).

### 5.5. Skin Diseases

Expression profiling has identified Ephx3 as a gene associated with ichthyosis [97], a congenital skin disorder characterized by hyperproliferation and defective epidermal barrier integrity. Functional studies in Ephx3 knockout mice confirm a barrier defect, as indicated by markedly increased transepidermal water loss despite a normal gross phenotype [98]. Biochemical analyses reveal reduced covalent binding of ceramides and an ~85% reduction in linoleate-derived trihydroxy isomers [99], implicating Ephx3 in the hydrolysis of lipid epoxides within the 12R-LOX/eLOX3 pathway. These findings establish Ephx3 as a critical enzymatic component for maintaining epidermal lipid architecture and barrier function. In addition, Ephx4 has been linked to sebaceous gland biology. The RNA interference–mediated knockdown of Ephx4 increases lipid droplet size and enhances sebaceous lipogenesis [100], suggesting that Ephx4 negatively regulates lipid accumulation in skin appendages.

### 5.6. Reproductive Disorders

Multiple genetic association studies implicate Ephx1 polymorphisms in pregnancy-related disorders [56]. Increased mEH-mediated EpFA hydrolysis has been associated with preeclampsia, with Ephx1 variants conferring elevated risk [101,102,103,104]. Similarly, increased sEH expression and activity correlate with preeclampsia [105,106], with elevated EpFA levels detected in the placenta, plasma, and urine of affected women.

In reproductive endocrinology, Ephx1 has also been linked to polycystic ovary syndrome (PCOS) [107]. Specific Ephx1 polymorphisms increase PCOS susceptibility, and reduced promoter methylation of EPHX1 in patients suggests the epigenetic activation of gene expression [108]. Elevated EPHX1 activity may impair androgen-to-estradiol conversion, thereby exacerbating hyperandrogenism and contributing to PCOS pathophysiology.

### 5.7. Inhibitors and Clinical Applications of the Epoxide Hydrolase Family

Among the EH family members, the development of inhibitors has been most extensively advanced for sEH, whereas studies on inhibitors targeting other EH members remain at an early stage. Previous studies have shown that methyl arachidonyl fluorophosphonateeffectively inhibits the hydrolysis of 2-arachidonoylglycerol in prostate cancer cells, thereby suppressing androgen-independent tumor cell invasion [109]. In addition, EPHX1 inhibitors, such as 10-hydroxystearamide, may exert protective effects by reducing the hydrolysis of endogenous epoxy fatty acids and endocannabinoids [110], leading to increased levels of bioactive lipid mediators and enhanced anti-inflammatory and tissue-protective signaling. These effects suggest potential therapeutic applications in inflammation, pain, and cardiovascular or neurological disorders.

However, because EPHX1 also plays an important role in the detoxification of exogenous epoxide compounds, its inhibition may impair xenobiotic metabolism and lead to the accumulation of toxic intermediates, thereby raising potential safety concerns. In contrast, the physiological functions and pharmacological significance of EPHX3 and EPHX4 remain incompletely defined. The current lack of selective inhibitors and clinical evidence limits the assessment of their therapeutic value, and further studies are required to establish their efficacy and safety profiles.

By comparison, sEH has emerged as the most promising therapeutic target within the EH family. Previous studies have demonstrated that sEH participates in the regulation of neuroinflammation and pain-related signaling through the hydrolysis of anti-inflammatory and neuroprotective epoxy fatty acids, particularly EETs [26,111,112]. The pharmacological inhibition of sEH stabilizes EET levels, thereby attenuating neuroinflammatory responses and modulating signaling pathways such as PI3K/Akt/GSK3β. These effects confer protective benefits in neurodegenerative diseases and inflammation-associated pain conditions. Accordingly, sEH is increasingly recognized as a critical molecular node linking lipid signaling metabolism with neuroinflammatory regulation and has gradually become a promising target for the development of novel non-opioid analgesics [26,111,112].

In recent years, the development of sEH inhibitors has progressed from basic research toward drug development. In clinical studies, the small-molecule sEH inhibitor EC5026 has entered human clinical trials as a non-opioid analgesic. Early studies indicate that EC5026 exhibits favorable tolerability and pharmacokinetic properties, providing a basis for further clinical investigation [113,114].

In preclinical research, multiple sEH inhibitors have demonstrated promising pharmacological activity in animal models. For example, AMHDU (1,1′-(hexane-1,6-diyl)bis(3-((adamantan-1-yl)methyl)urea)) exhibits potent analgesic effects in neuropathic pain models. In a diabetic neuropathic pain model, a 10 mg/kg dose significantly increases pain thresholds and alleviates tactile allodynia, with efficacy comparable to gabapentin. Moreover, AMHDU shows low acute oral toxicity (>2000 mg/kg) and a high therapeutic index, indicating its potential for further structural optimization and preclinical development [115]. Overall, these findings suggest that small-molecule sEH inhibitors hold promise for the treatment of pain and inflammation-related disorders and provide experimental support for the further development of sEH-targeted therapeutics [113,115].

## 6. Discussion

EHs represent a versatile enzyme family that integrates xenobiotic detoxification, lipid metabolism, and redox regulation, thereby shaping both physiological homeostasis and pathological processes [116]. In this review, we highlight how EH isoforms orchestrate cellular and systemic responses across diverse contexts, ranging from chemical defense to inflammation and organ-specific disease mechanisms. While EPHX1 and EPHX2 have been extensively investigated as canonical members, emerging evidence on EPHX3 and EPHX4 expands the functional landscape of this enzyme family and underscores its evolutionary and mechanistic diversity.

A recurring theme is the dualistic role of EHs in health and disease [117]. On the one hand, mEHs safeguard cells from toxic epoxides and sEHs modulate fatty acid-derived lipid mediators that critically influence cardiovascular, hepatic, and neuroinflammatory pathways [118]. On the other hand, dysregulated EH activity perturbs lipid signaling, amplifies inflammatory cascades, and contributes to the pathogenesis of complex disorders such as Alzheimer’s disease, cancer, and metabolic syndromes [83,86,94]. It is noteworthy that, in addition to EETs derived from arachidonic acid (ARA), other polyunsaturated fatty acids (PUFAs) can also be converted into various EpFAs via the cytochrome P450 (CYP450) epoxygenase pathway. These lipid mediators are further hydrolyzed by sEH into their corresponding diol products [119]. Studies have demonstrated that these PUFA-derived EpFAs play significant biological roles in the regulation of inflammation, pain signaling, angiogenesis, and tumor development, with their effects being closely associated with sEH-mediated metabolism [120,121]. However, this review primarily focuses on ARA-derived EETs and DHETs, with limited coverage of the sEH-mediated regulation and functions of EpFAs derived from other PUFAs, which constitutes a limitation of the present work. Future studies are warranted to systematically summarize and explore the sEH-mediated metabolism of these lipid signaling molecules and their biological significance. Despite these advances, substantial gaps remain in defining isoform-specific substrate spectra, regulatory mechanisms, and spatiotemporal dynamics. Most current knowledge derives from rodent models or in vitro studies, with limited validation in human tissues or clinical cohorts. This translational gap obscures how EHs function in the intricate milieu of human disease.

Another challenge lies in therapeutic targeting. The pharmacological inhibition of sEH has yielded promising results in preclinical models of cardiovascular, neurodegenerative, and psychiatric disorders. Nevertheless, long-term suppression of EH activity raises safety concerns, given its essential roles in xenobiotic metabolism [15,34]. Off-target effects on detoxification pathways may offset therapeutic benefits, emphasizing the need for highly selective, context-dependent modulators and the rigorous evaluation of toxicity profiles.

Looking forward, several research directions are particularly compelling. High-throughput omics technologies—including lipidomics, single-cell transcriptomics, and epigenomic profiling—could unravel the substrate networks and regulatory circuits of less-studied isoforms such as EPHX3 and EPHX4. Comparative and cross-species analyses may illuminate evolutionary conservation versus divergence, offering clues to functional specialization. In addition, the integration of environmental exposures into experimental frameworks may reveal how EHs interface with epigenetic reprogramming and metabolic adaptation, thereby linking gene–environment interactions to disease susceptibility.

EHs have emerged as pivotal regulators at the interface of detoxification, lipid signaling, and inflammation. Yet, isoform-specific functions [122], context-dependent regulation [54], and translational relevance remain only partially defined. Bridging mechanistic insights with human biology, and integrating omics-based discovery with therapeutic innovation, will be essential to unlock the full potential of this enzyme family. Ultimately, deciphering how EHs govern metabolic and inflammatory circuits may open new avenues for precision medicine across a broad spectrum of human diseases.

## 7. Conclusions

The EH family is widely expressed and participates in diverse biological processes, from detoxification to lipid signaling and inflammation. Elucidating their mechanistic roles is crucial for unraveling the pathophysiology of complex diseases and for advancing therapeutic strategies targeting EHs. By systematically summarizing their structures, functions, and disease associations, this review provides a foundation for the deeper exploration of EH biology. Future work integrating molecular, omics, and translational approaches will be essential to fully harness the potential of EHs as diagnostic markers and therapeutic targets.

## Figures and Tables

**Table 1:** Roles of EH family genes in human cancers.

Cancer Type	EH Members	Key Findings	Function/Clinical Significance
Laryngeal Squamous Cell Carcinoma (LSCC)	Ephx4	Elevated Ephx4 expression promotes tumor proliferation and migration; patients with low expression exhibit longer survival and increased immune cell infiltration.	Ephx4 is a potential adverse prognostic marker and therapeutic.
Renal and Hepatic Carcinomas	Ephx4	Ephx4 is upregulated in clear cell renal cell carcinoma, papillary renal cell carcinoma, and hepatocellular carcinoma.	Ephx4 may serve as a potential drug target and prognostic biomarker.
Colorectal Cancer (CRC)	Ephx1/2/3/4	Downregulation of Ephx2 inhibits CRC cell invasion; Ephx4 overexpression promotes cellular transformation; Ephx3 is upregulated; Ephx1 is downregulated.	Ephx2 functions as a tumor suppressor, Ephx4 promotes tumor progression, and Ephx3 may serve as a diagnostic biomarker.
Head and Neck SCC (HNSCC)	Ephx3	High Ephx3 expression is associated with hypomethylation and improved survival; miRNA-mediated downregulation of Ephx3 suppresses tumor proliferation.	Ephx3 is a potential favorable prognostic biomarker.
Lung Cancer	Ephx1	mEH polymorphisms influence lung cancer susceptibility; Y113H is protective, whereas H139R is associated with increased risk.	Ephx1 polymorphisms are associated with lung cancer risk.
Prostate Cancer (PCa)	Ephx2	Reduced Ephx2 expression is associated with PCa progression and poor prognosis, with limited effects on cell proliferation and antiandrogen therapy response.	Ephx2 may serve as a potential prognostic biomarker.
Other Cancers	Ephx1/3	Ephx1 polymorphisms are implicated in esophageal, bladder, and breast cancers; ABHD9 hypermethylation is observed in multiple tumor types.	Ephx1 is associated with susceptibility across multiple cancers, and ABHD9 methylation may represent a novel therapeutic target.

Abbreviations: LSCC: laryngeal squamous cell carcinoma; HNSCC: head and neck squamous cell carcinoma; CRC: colorectal cancer; PCa: prostate cancer.

## Data Availability

No data was used for the research described in the article.

## Abbreviations

EHs	Epoxide hydrolases
EpFA	Epoxy fatty acid
mEH	Microsomal EH
EETs	Epoxy eicosatrienoic acids
sEH	Soluble EH
AD	Alzheimer’s disease
PTMs	Post-translational modifications
EREs	Estrogen Response Elements
SULT	Sulfur transferase
UGT	UDP-glucuronic acid transferase
ER	Estrogen receptor
ROS	Reactive oxygen species
LSCC	Laryngeal squamous cell carcinoma
ALD	Alcohol-related liver disease
AKI	Acute kidney injury
CKD	Chronic kidney disease
EMT	Epithelial–mesenchymal transition
DHETs	Dihydroxy eicosatrienoic acids
PAHs	Polycyclic aromatic hydrocarbons
VSMC	Vascular smooth muscle cell
APP	Amyloid precursor protein
TBI	Traumatic brain injury
CCI	Controlled cortical impact
CHI	Closed-head injury
PCOS	Polycystic ovary syndrome
ARA	Arachidonic acid
PUFAs	Polyunsaturated fatty acids
